# Surface-Modified Nanozymes for Enhanced and Selective Catalysis

**DOI:** 10.1021/acsami.5c07647

**Published:** 2025-07-23

**Authors:** Xinghua Chen, Itamar Willner

**Affiliations:** Institute of Chemistry, The Hebrew University of Jerusalem, Jerusalem 91904, Israel

**Keywords:** Nanoparticle, Aptamer, Molecular-imprinted polymer, Chiroselectivity, Nanomedicine, Reactive oxygen species (ROS)

## Abstract

Surface-modified catalytic nanoparticles (nanozymes) are introduced as hybrid nanoparticles overcoming basic limitations associated with bare nanozymes that include moderate catalytic turnovers, lack of substrate selectivity and chiroselectivity, and poor or nonselective permeabilities into biomembrane. This review introduces aptamer-modified nanozymes, receptor (cyclodextrins)- or ligand (amino acids, peptides)-functionalized catalytic nanoparticles, and molecularly imprinted polymer-coated nanozymes as hybrid frameworks improving the catalytic properties and selective/chiroselective functions of the nanozymes. Binding of the reaction substrates to the aptamers, ligands, or molecular-imprinted sites, by affinity interactions, concentrates the substrates in spatial proximity to the nanozyme catalytic sites (“molarity effect”), thereby enhancing the catalytic performance of the frameworks. Specific and chiroselective binding interactions of the substrates to the surface modifiers lead to selective or chiroselective chemical transformations. Moreover, by appropriate molecular engineering of the surface modifiers on the nanozymes, catalytic functions lacking in the parent bare nanozymes are demonstrated. Potential applications of surface-modified nanozymes are discussed.

## Graphical abstract

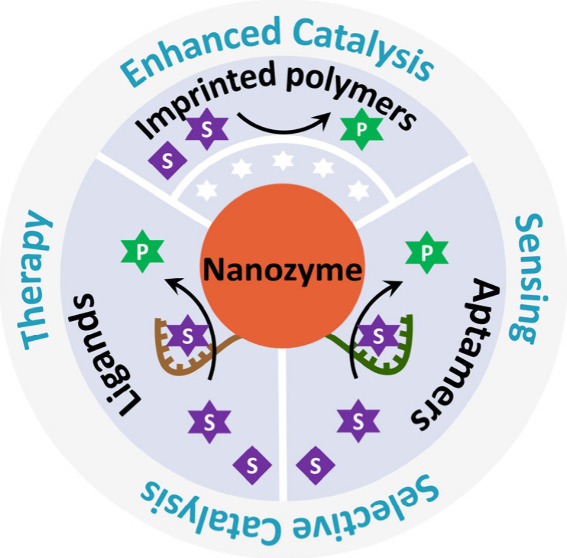

## 1 Introduction

Substantial recent research efforts have been directed to the development of synthetic inorganic, organic, or composite organic–inorganic nanoparticles and nanostructures emulating native enzyme functions, nanozymes.^1–7^ Inorganic metal particles, such as Pt,^8^ Pd,^9^ Au,^10^ Ru,^11,12^ metal oxides, e.g., Fe_3_O_4_,^13^ CeO_2_,^14^ MoO_3_,^15^ metal alloys^16,17^ or carbon-based nanomaterials, such as carbon dots,^18,19^ graphene^20^ or graphene oxide,^21,22^ polymer nanoparticles, such as polydopamine,^23,24^ and metal–organic frameworks^25–27^ or covalent-organic frameworks^28–30^ have been used as catalytic nanozyme structures. Diverse chemical transformations mimicking native enzymes or driving non-natural chemical transformations have been demonstrated. These included redox reactions,^31–34^ hydrolysis reactions,^35–38^ and lysis reactions.^39,40^ Diverse applications of nanozymes including the development of sensors,^41–44^ imaging agents,^45–48^ medical applications,^49–51^ degradation of pollutants,^52–54^ and water purification^55,56^ were reported. While nanozymes reveal advantages as compared to native enzymes, reflected by high stabilities under harsh conditions, operation in aqueous and organic solvents, scalability, and cost effectiveness, nanozymes also suffer from limitations as compared to their biological counterparts. These include limited catalytic turnover as compared to native enzymes, lack of substrate specificity, and chiroselectivity. These drawbacks are mainly due to the evolutionarily optimized structures of native enzymes that include complex tertiary programmed chiral folded protein structures and supramolecular engineered active site environments. These features lead to high-affinity and chiroselective binding of the reaction substrates, allosteric interactions, and cooperative catalytic functions of the active site environments. These significant features are missing in synthetic nanozyme frameworks. Thus, efforts to enhance the structural and functional complexity of nanozymes are essential to improve their catalytic functions.

In fact, many of the nanozyme surfaces can adsorb or covalently link molecular ligands or chemically modified monolayers. Although such molecular adsorbents often perturb the catalytic sites associated with the nanozymes, they might be used as functional anchoring sites for the tethering of cooperative catalytic sites or chiral ligands in spatial proximity to the nanozyme catalytic sites. These can cooperatively activate the nanozyme catalytic sites, provide chiral environments, and eventually allow the synthesis of nanozyme/ligand hybrid conjugates emulating the features of enzyme active sites. Moreover, many of the hydrothermal, sonochemical, or microwave procedures synthesizing nanozymes from organic or inorganic precursors yield nanoparticles with surface functionalities, such as −OH, −NH_2_, and −COOH. These surface functionalities provide ligation sites for diverse metal ions, transition complexes, and chiral ligands or anchoring sites for binding reaction substrates in spatial proximity to the nanozyme frameworks. Indeed, surface modification of nanozymes with molecular, macromolecular, or biomaterial agents provides versatile means to enhance the catalytic activities of nanozymes through concentration of the substrates at the catalytic interfaces^57,58^ and to increase the selectivity and chiroselectivity of nanozymes by dictated affinity interaction between the modifying ligands and the reaction substrates through electrostatic interactions,^59^ H-bonds,^60,61^ host–guest complexes,^62,63^ and molecular imprinted sites.^64,65^ Moreover, the surface modifying ligands may act as cell targeting means improving cell recognition and tissue permeation.^66,67^ These beneficial features of surface-modified nanozymes find broad applications of the hybrid frameworks for enhanced, selective catalysis,^68^ analytical sensing and imaging applications,^69^ and advanced therapeutic methods.^70^ The present review summarizes the efforts to modify nanoparticle frameworks with substrate binding ligands, polymers, and chiral environments generating nanozymes of superior catalytic properties. The applications of these nanozymes for sensing and medical applications are discussed.

## 2 Oligonucleotide-Modified Nanozymes

The base sequences encoded in DNA-based synthetic strands may be programmed to reveal dictated and specific recognition functions toward low-molecular-weight ligands or macromolecules (aptamer).^71,72^ These sequence-specific strands are elicited, selected, and amplified by the systematic evolution of ligands by an exponential enrichment (SELEX) process.^73–76^ These unique binding properties of aptamers have found diverse applications as sensing elements,^77–80^ therapeutic agents through inhibition of proteins^81–83^ and specific drug carriers,^84–87^ intracellular imaging agents of biomarkers,^88–90^ and self-organization of catalytic agents.^91^ Moreover, conjugation of aptamer units to catalytic DNA strands, e.g., hemin/G-quadruplex,^92^ or coupling of aptamers to homogeneous catalysts such as Cu^2+^-terpyridine or Zn^2+^-salen complexes,^93–95^ yielded catalytic agents (“nucleoapzyme”) mimicking the binding/catalytic site functions of native enzymes. These approaches were extended to conjugate aptamer units to nanozyme agents to yield hybrid “aptananozyme” frameworks revealing specific substrate binding functions in spatial proximity to the catalytic sites of nanozymes, thereby emulating the recognition properties and catalytic functions of active sites of native biocatalysts.^57^ These cooperative functions of aptananozymes could, then, be implemented for targeted, enhanced (concentration of substrate—“molarity effect”), and selective catalysis.

This is exemplified in Figure 1A with the use of antidopamine aptamer-functionalized Cu^2+^-modified carbon dots (Cu^2+^-C-dots) as aptananozyme frameworks.^57^ The Cu^2+^-C-dots were functionalized with a library of amino-aptamer strands i–v consisting of antidopamine aptamer sequences and different (TGTA) spacing bridges (Figure 1B, Panel I). All aptananozymes revealed enhanced catalytic activities toward the catalyzed oxidation of dopamine to aminochrome, as compared to the system consisting of the separated Cu^2+^-C-dots/aptamer, while the conjugate iv demonstrated 50-fold enhancement toward the oxidation of dopamine (Figure 1B, Panel II). These results are consistent with the concentration of the dopamine substrate (“molarity effect”) in the spatial proximity of the catalytic sites. This conceptual approach was further developed to construct a superior aptananozyme for the catalyzed H_2_O_2_ oxidation of l-tyrosinamide to amidodopachrome by conjugation of the anti-l-tyrosinamide aptamer to the Cu^2+^-C-dots.^57^ Moreover, the antidiabetic aptamer-modified Cu^2+^-C-dots represent chiral frameworks due to the chirality associated with the aptamer binding sites. Accordingly, the aptananozyme is anticipated to reveal chiral selectivity toward the catalyzed oxidation of chiral substrates. Indeed, the chiroselective oxidation of l-/d-DOPA to dopachrome in the presence of H_2_O_2_ was demonstrated (Figure 1B, Panel III). The oxidation of l-DOPA to dopachrome was 2-fold enhanced as compared to the oxidation of d-DOPA to dopachrome, consistent with the higher binding affinity of l-DOPA as compared to d-DOPA to the chiral aptamer (*K*_d,_ l‑DOPA= 1.7 μM, *K*_d,_ d‑DOPA = 6.6 μM). In a related study,^58^ Ce^4+^-modified C-dots (Ce^4+^-C-dots) functionalized with the antidopamine aptamer units were employed as aptananozymes for the catalyzed H_2_O_2_ oxidation of dopamine to aminochrome. Similarly, the concentration of the dopamine substrate by the aptamer units in spatial proximity to the Ce^4+^-C-dots catalytic interface led to enhanced catalytic oxidation of dopamine to aminochrome, compared to the separated Ce^4+^-C-dots/aptamer constituents. The superior aptananozyme consisting of the antidopamine aptamer linked to the catalytic nanozyme interface through a 2×(TGTA) bridge demonstrated a 14-fold enhanced oxidation rate of dopamine to aminochrome, as compared to the separated constituents. Mechanistic studies demonstrated that the reactive oxygen species (ROS), hydroxyl radical (^•^OH), generated by the interaction of the catalytic nanozyme, Ce^4+^-C-dots, with H_2_O_2_ participated in the oxidation of dopamine to aminochrome (Figure 1C, Panel I). The spatial proximity between the catalytically generated ^•^OH and the aptamer-stimulated concentration of the reactive substrate at the aptananozyme interface resulted in the effective oxidation process (Figure 1C, Panel II).

**Figure 1:**
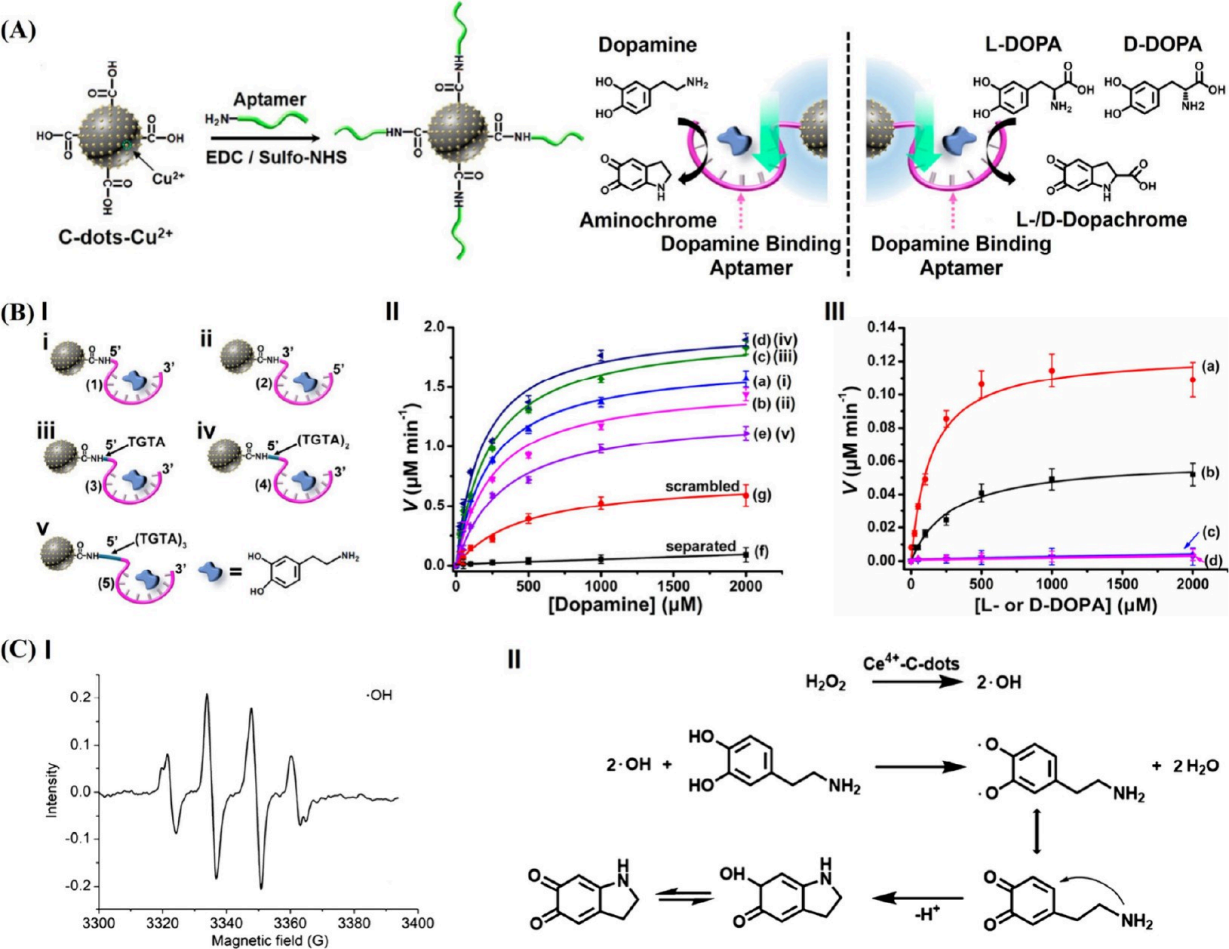
(A) Aptamer-modified Cu^2+^-C-dots aptananozyme for the catalyzed H_2_O_2_ oxidation of dopamine to aminochrome or the chiroselective oxidation of l-/d-DOPA to aminochrome. (B) Panel I: a set of configurations of dopamine aptamer-functionalized Cu^2+^-C-dots aptananozymes. Panel II: rates of dopamine oxidation by H_2_O_2_ in the presence of the set of aptananozymes shown in Panel I, as a function of dopamine concentrations. Panel III: rates of chiroselective oxidation of l-/d-DOPA with H_2_O_2_ by aptananozyme-i as compared to the separated Cu^2+^-C-dots and dopamine aptamer in the presence of various concentrations of l-/d-DOPA ((a) oxidation of l-DOPA by the aptananozyme-i; (b) oxidation of d-DOPA by the aptananozyme-i; (c, d) oxidation of l-DOPA (c) or d-DOPA (d) by the separated Cu^2+^-C-dots and dopamine aptamer). Reproduced from ref 57. Available under a CC-BY 4.0 license. Copyright 2021 ACS. (C) Panel I: electron spin resonance spectrum of the hydroxyl radical (^•^OH) generated by the dopamine aptamer-modified Ce^4+^-C-dots in the presence of H_2_O_2_. Panel II: mechanistic pathway corresponding to the dopamine aptamer-modified Ce^4+^-C-dots aptananozyme catalyzing the H_2_O_2_ oxidation of dopamine to aminochrome. Reproduced from ref 58. Available under a CC-BY 4.0 license. Copyright 2022 ACS.

As sequence-specific aptamers bind to cancer cell receptors, and ROS act as cytotoxic agents toward cancer cells, aptananozymes consisting of cancer cell targeting aptamers conjugated to ROS generating nanozymes were proposed as selective catalysts for chemodynamic treatment of cancer cells.^58^ This is exemplified in Figure 2A with the synthesis of antinucleolin aptamer (AS1411)- or anti-MUC-1 aptamer-functionalized Ce^4+^-C-dots and their use for the chemodynamic treatment of cancer cells. The recognition functions of AS1411 or anti-MUC-1 aptamer toward nucleolin or MUC-1 receptors associated with MDA-MB-231 breast cancer cells favored the localization of the Ce^4+^-C-dots to the cancer cells, thereby catalyzing the generation of ROS agents for the targeted chemodynamic treatment of MDA-MB-231 breast cancer cells and tumors. The AS1411 or MUC-1 aptamer-modified nanozymes caused ca. 90% cell death toward MDA-MB-231 breast cancer cells and ca. 10% cell death of normal epithelial cells, revealing significant selectivity (Figure 2B, Panel I). Moreover, the aptananozymes reflected efficient inhibition of the tumor growth (>85%), as compared to that treated with nonaptamer-modified nanozyme (Figure 2B, Panel II). In a related study,^66^ polyadenine (pA)-stabilized catalytic Au nanoparticles, appended with the AS1411 aptamer, acted as an aptananozyme bioreactor for the selective *in vitro* and *in vivo* chemodynamic treatment of MDA-MB-231 breast cancer cells and tumors through intracellular glucose-driven generation of ROS agents (Figure 2C). The Au nanoparticles exhibit glucose oxidase-like activity catalyzing the aerobic oxidation of glucose to gluconic acid and H_2_O_2_ and peroxidase-like activity transforming the generated H_2_O_2_ into cytotoxic ROS agents for the selective chemodynamic treatment of cancer cells utilizing the targeting function of the associated AS1411 aptamer. Indeed, an obvious apoptosis of MDA-MB-231 breast cancer cells (ca. 70%) was observed upon treatment with the aptananozyme, as compared to the unaffected cell death of normal epithelial MCF-10A breast cells, treated with similar conditions (Figure 2D, Panel I). Similarly, the aptananozyme-treated MDA-MB-231 tumor was inhibited by ca. 65%, while tumors treated with the nanozyme lacking AS1411 aptamer were not inhibited (Figure 2D, Panel II).

**Figure 2:**
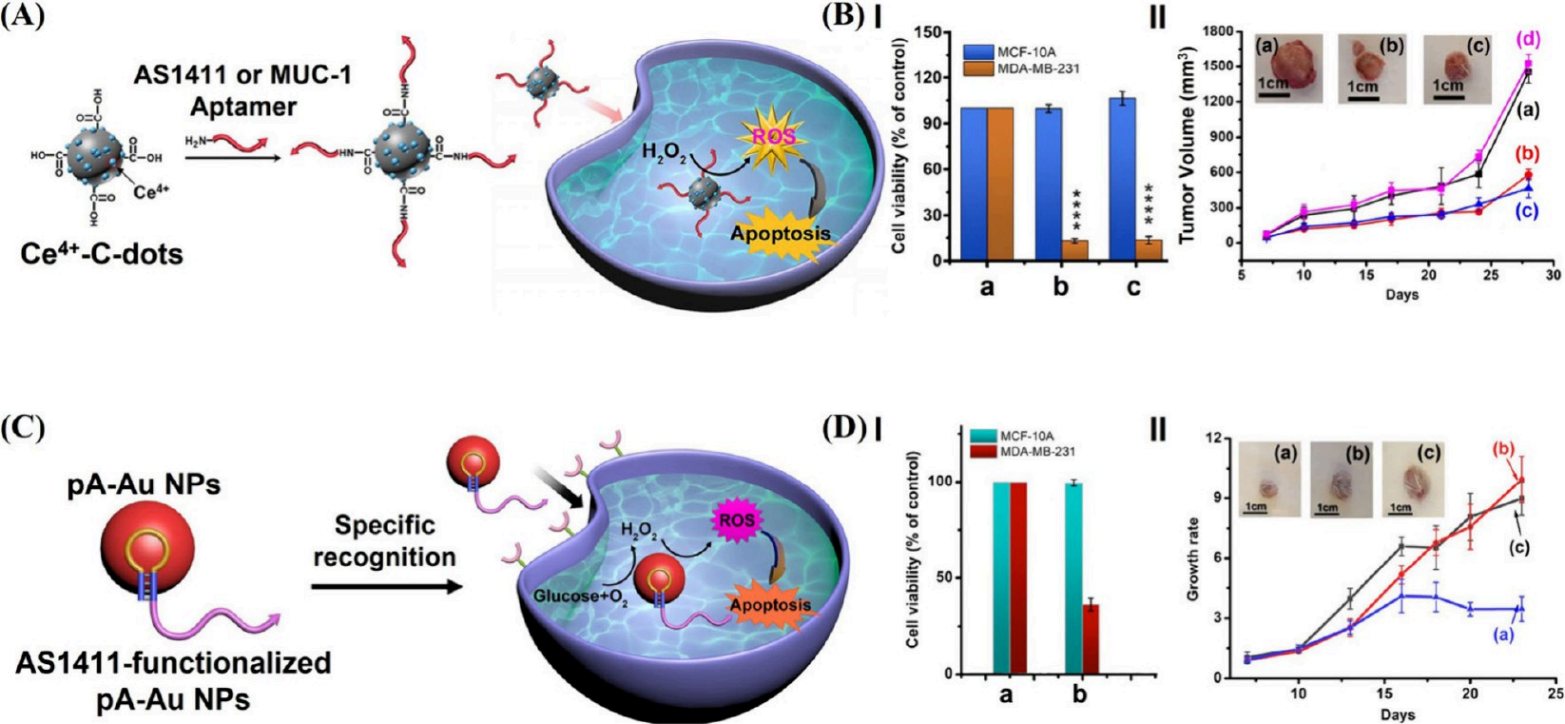
(A) Schematic ROS-driven apoptosis of cancer cells using the AS1411 aptamer or MUC-1 aptamer-modified Ce^4+^-C-dots as ROS generating agent. (B) Panel I: cell viability of MDA-MB-231 breast cancer cells and MCF-10A epithelial breast cells ((a) nontreated control cells; (b) treated with AS1411 aptamer-modified Ce^4+^-C-dots; (c) treated with MUC-1 aptamer-modified Ce^4+^-C-dots). Panel II: temporal MDA-MB-231 tumor growth treated with (a) Ce^4+^-C-dots, (b) AS1411 aptamer-modified Ce^4+^-C-dots, (c) MUC-1 aptamer-modified Ce^4+^-C-dots, and (d) control PBS buffer. Reproduced from ref 58. Available under a CC-BY 4.0 license. Copyright 2022 ACS. (C) Schematic ROS-driven apoptosis of cancer cells using Au NPs stabilized with polyadenine/AS1411 aptamer strand as bioreactor generating ROS agent. (D) Panel I: cell viability of MDA-MB-231 breast cancer cells and MCF-10A epithelial breast cells ((a) nontreated control cells; (b) treated with AS1411 aptamer-expanded polyadenine-stabilized Au NPs). Panel II: temporal MDA-MB-231 tumor growth treated with (a) random sequence-expanded polyadenine-stabilized Au NPs, (b) polyadenine-stabilized Au NPs, and (c) AS1411 aptamer-extended polyadenine-stabilized Au NPs. Reproduced from ref 66. Available under a CC-BY 4.0 license. Copyright 2022 ACS.

Moreover, stabilization of Au nanoparticles with chiral single-strand DNA or DNA structures such as duplex, G-quadruplex, or i-motif systems yielded Au nanozymes revealing chiroselective oxidation of l-/d-glucose.^96^ The Au nanoparticles exhibit glucose oxidase-like activity, thus allowing the operation of the chiroselective catalytic cascade where glucose is aerobically oxidized to gluconic acid and H_2_O_2_, and the resulting H_2_O_2_ oxidizes in the presence of horseradish peroxidase (HRP) 2,2′-azino-bis­(3-ethylbenzothiazoline-6-sulfonic acid) (ABTS^2–^) to the colored product ABTS^–•^ (Figure 3A). Interestingly, single-strand DNA-coated Au nanoparticles led to enhanced chiroselective oxidation of l-glucose as compared to d-glucose (Figure 3B, Panel I). In contrast, functionalization of Au nanoparticles by duplex DNA favored the chiroselective oxidation of d-glucose as compared to l-glucose (Figure 3B, Panel II). The chiral selectivity was found to be controlled by the concentration of the DNA modifiers coating the particles (Figure 3B, Panel III).

**Figure 3:**
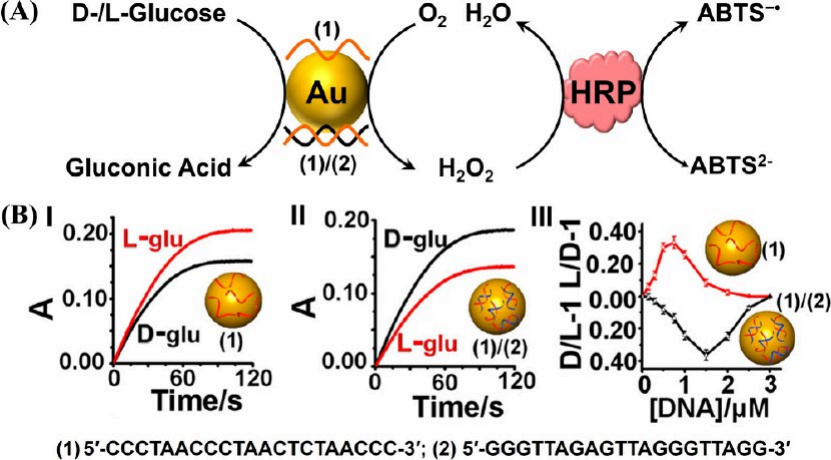
(A) Schematic single strand (**1**) or duplex nucleic acid (**1**)/(**2**)-modified Au NPs for the chiroselective aerobic oxidation of l-/d-glucose to gluconic acid and H_2_O_2_, probed by analyzing the resulting H_2_O_2_ by the secondary HRP-catalyzed oxidation of ABTS^2–^ to the colored ABTS^–•^. (B) Chiroselective aerobic oxidation of l-/d-glucose catalyzed by the **(1)**-modified Au NPs (Panel I) and **(1)**/**(2)**-modified Au NPs (Panel II). Panel III: chiroselective aerobic oxidation of l-/d-glucose by the **(1)**- or **(1)**/**(2)**-modified Au NPs as a function of the concentration of the nucleic acid modifiers used to coat Au NPs. Reproduced from ref 96. Copyright 2015 ACS.

## 3 Cyclodextrin-, Amino Acid-, and Peptide-Modified Nanozymes

Chemical modification of nanozymes with surface-bound ligands has been used to stabilize nanoparticles, enhance their solution suspendability, promote the transfer capability from aqueous phase into oleophilic phases, and improve permeability into cellular membranes. The surface modification of catalytic nanoparticles is, however, often hampered by the deactivation of the nanozymes due to the blockage of catalytic sites by the surface modifying ligands. Nevertheless, modification of the nanozyme surfaces with receptor ligands interacting with the nanozyme substrates may increase the local concentration of the substrates at the catalytic interfaces, thereby enhancing the nanozyme activities. Alternatively, coating the nanozymes with chiral ligands may provide chiral environments at the catalytic sites, thereby inducing chiroselective nanozyme-driven catalytic transformations.

Cyclodextrins are cyclic frameworks composed of glucose units interlinked by 1,4-glycosidic bonds.^97,98^ The cyclodextrin frameworks form cone-shaped water-soluble structures composed of hydrophobic cavities functionalized at their upper and lower rings with hydroxyl groups. The size of the cyclodextrin cavities is controlled by the number of interlinked glucose units composing the cyclic receptors (e.g., β-cyclodextrin, β-CD, is composed of seven interlinked glucose units). Cyclodextrins have found diverse applications including separation,^99,100^ sensing,^101,102^ and stabilization of hydrophobic agents in aqueous environment through binding to the framework cavities^103^ and enhancing catalytic functions through chemical modification of the cyclodextrin receptor units.^104,105^ Moreover, the chiral features of the glucose units composing the cyclodextrins have been implemented for chiral separation and chiroselective reactions.^106,107^ Diverse applications of the selective or chiroselective binding functions of cyclodextrin were reported including drug delivery,^108,109^ food industries,^110^ and synthesis of stimuli-responsive “smart” materials (e.g., hydrogels of controlled stiffness).^111,112^

Indeed, cyclodextrin receptors associated with nanozyme particles provide a versatile means to bind and concentrate the reaction substrates in the cyclodextrin cavities in spatial proximity to the nanozyme catalytic interfaces. Figure 4A exemplifies the synthesis of a β-CD-modified Cu^2+^-C-dots nanozyme for the enhanced catalytic oxidation of dopamine to aminochrome.^113^ Amino-modified β-CD was covalently linked to surface carboxylic acid groups associated with the Cu^2+^-C-dots. The binding of dopamine to the β-CD concentrated the reaction substrate at the nanozyme surfaces, resulting in a 4-fold enhanced oxidation of dopamine to aminochrome as compared to the Cu^2+^-C-dots nanozyme (Figure 4B). A related study^63^ demonstrated the stabilization of Pd@Au nanoparticles by β-CD (Figure 4C). The β-CD-stabilized particles revealed peroxidase-like activity catalyzing the H_2_O_2_ oxidation of 3,3′,5,5′-tetramethylbenzidine (TMB) to the colored TMB^+•^ (λ = 652 nm) through the intermediate generation of ^•^OH radicals. The concentration of TMB in the β-CD cavities then enhanced the oxidation rate of TMB by H_2_O_2_ (Figure 4D). The nanozyme-amplified H_2_O_2_ oxidation of TMB to TMB^+•^ by the β-CD-modified Pd@Au particles was further applied to develop a colorimetric glucose sensor (Figure 4E). The glucose oxidase (GOx) catalyzes the aerobic oxidation of glucose to gluconic acid and H_2_O_2_, and the resulting H_2_O_2_ activates the cascaded Pd@Au nanozyme-catalyzed oxidation of TMB to TMB^+•^. As the concentration of the resulting H_2_O_2_ is controlled by the concentration of glucose, the resulting absorbance of TMB^+•^ provides a quantitative signal for the colorimetric detection of glucose, as shown in Figure 4F.

**Figure 4:**
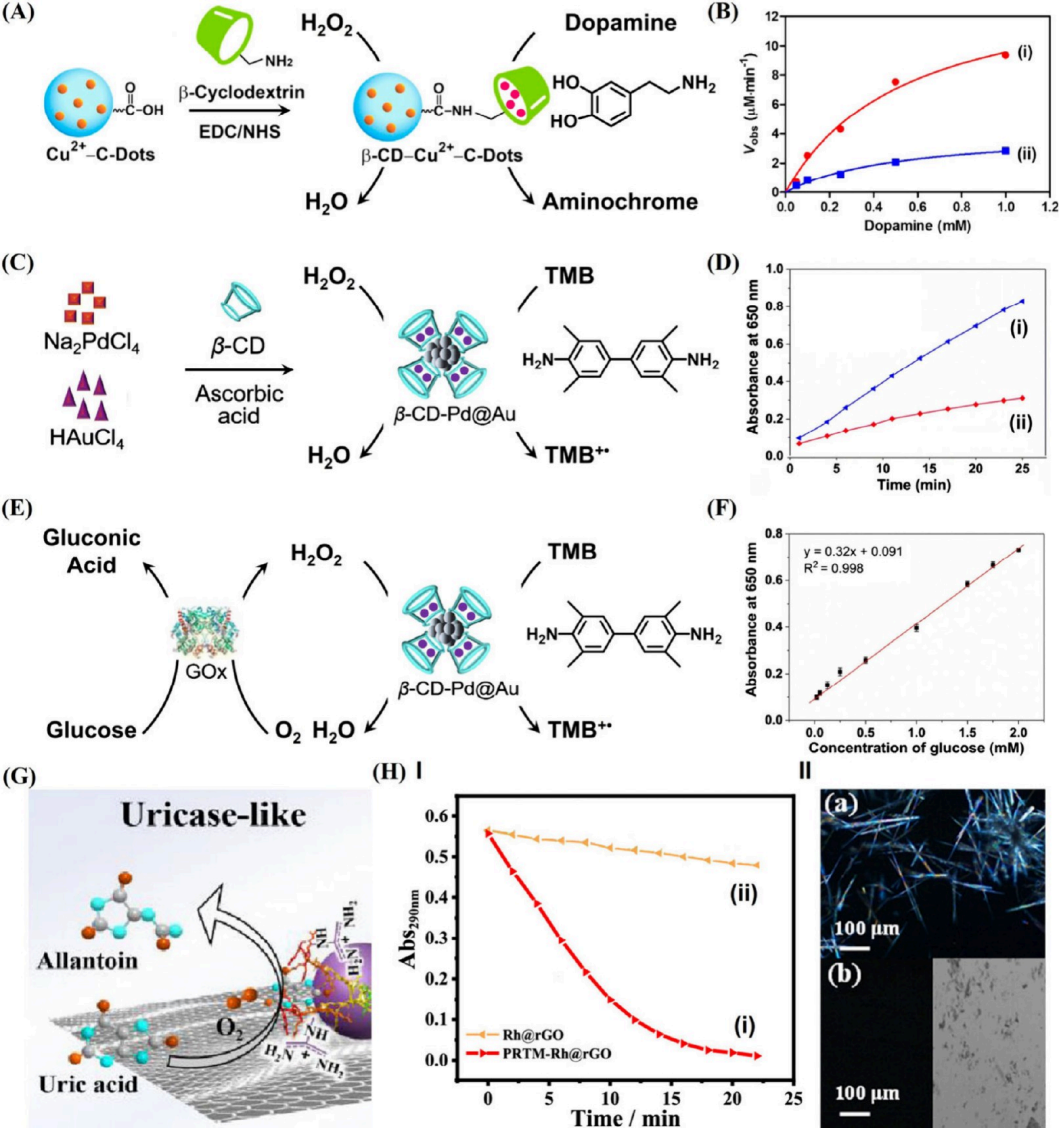
(A) β-CD-modified Cu^2+^-C-dots as a receptor-modified nanozyme for enhanced oxidation of dopamine by H_2_O_2_. (B) Rates of dopamine oxidation by H_2_O_2_ at different concentrations of dopamine: (i) in the presence of the integrated β-CD/Cu^2+^-C-dots nanozyme; (ii) in the presence of nonmodified Cu^2+^-C-dots. Reproduced from ref 113. Copyright 2017 ACS. (C) Synthesis of β-CD-modified Pd@Au NPs for the enhanced oxidation of TMB. (D) Time-dependent absorbance changes upon oxidation of TMB to TMB^+•^ by H_2_O_2_: (i) in the presence of β-CD-modified Pd@Au NPs; (ii) in the presence of bare Pd@Au NPs. (E) Application of the β-CD-modified Pd@Au NPs for the colorimetric detection of glucose. (F) Calibration curve relating the absorbance changes of the colorimetric probe (TMB^+•^) to variable concentrations of glucose, sensed by the β-CD-modified Pd@Au NPs. Reproduced with permission from ref 63. Copyright 2020 Springer Nature. (G) Protamine-functionalized Rh/rGO acting as a hybrid nanozyme for the aerobic oxidation of uric acid to allantoin. (H) Panel I: time-dependent absorbance changes upon aerobic oxidation of uric acid by (i) the protamine-functionalized Rh/rGO and (ii) the bare Rh/rGO. Panel II: optical micrograph corresponding to (a) the nontreated urate crystal and (b) the urate crystal treated with protamine-functionalized Rh/rGO. Reproduced from ref 114. Copyright 2024 ACS.

Alternatively, coating of the nanozymes with oligopeptides provided affinity binding interfaces concentrating the substrates in proximity of the catalytic sites by electrostatic, H-bonding, or supramolecular donor–acceptor affinity interactions. The use of oligopeptides as functional interface concentrating the reaction substrate at the catalytic nanozyme framework is depicted in Figure 4G with the assembly of an effective nanozyme assembly emulating the catalytic function of uricase.^114^ Rhodium (Rh) nanoclusters deposited on reduced graphene oxide (rGO) were coated with the arginine-rich peptide protamine. The resulting composite revealed uricase-like activity demonstrated by the effective aerobic oxidation of uric acid, UA, to allantoin (Figure 4H). The oxidation of UA to allantoin was ca. 42-fold enhanced in the presence of the rGO-supported peptide-coupled Rh clusters, as compared with the bare Rh clusters/rGO composites. This rate enhancement was attributed to the affinity binding of UA to the peptide coating via cooperative electrostatic and H-bond interactions. In fact, the aerobic oxidation of UA by the oligopeptide/Rh/rGO nanozyme is important for potential medical applications, as the deposition of UA crystalline in peripheral joints and tissues leads to inflammatory arthritis.^115^ The catalytic degradation of UA offers an effective therapeutic approach for treating this condition.^116,117^ Indeed, Figure 4H demonstrated that peptide-coated Rh/rGO effectively prohibited the crystallization of UA.

The availability of pure l- or d-amino acids makes these chemicals ideal ligands to modify nanozymes while generating nanocatalysts with opposite chirality. This is exemplified in Figure 5(A) with the functionalization of SiO_2_-immobilized Au NPs with l- or d-cysteine (l-/d-Cys) ligands.^60^ The resulting l-Cys-Au NPs or d-Cys-Au NPs exhibited peroxidase-like activity, catalyzing the oxidation of l-/d-DOPA to l-/d-dopachrome by H_2_O_2_. Chiroselective oxidation of l-/d-DOPA to l-/d-dopachrome by H_2_O_2_ in the presence of the chiral nanozymes was demonstrated. While l-Cys-Au NPs revealed a 1.7-fold enhanced oxidation rate of D-DOPA to d-dopachrome, as compared to l-DOPA, opposite chiroselectivity was observed with the d-Cys-Au NPs, where a 1.5-fold enhancement of oxidation rate of l-DOPA to l-dopachrome as compared to d-DOPA was demonstrated (Figure 5B).

**Figure 5:**
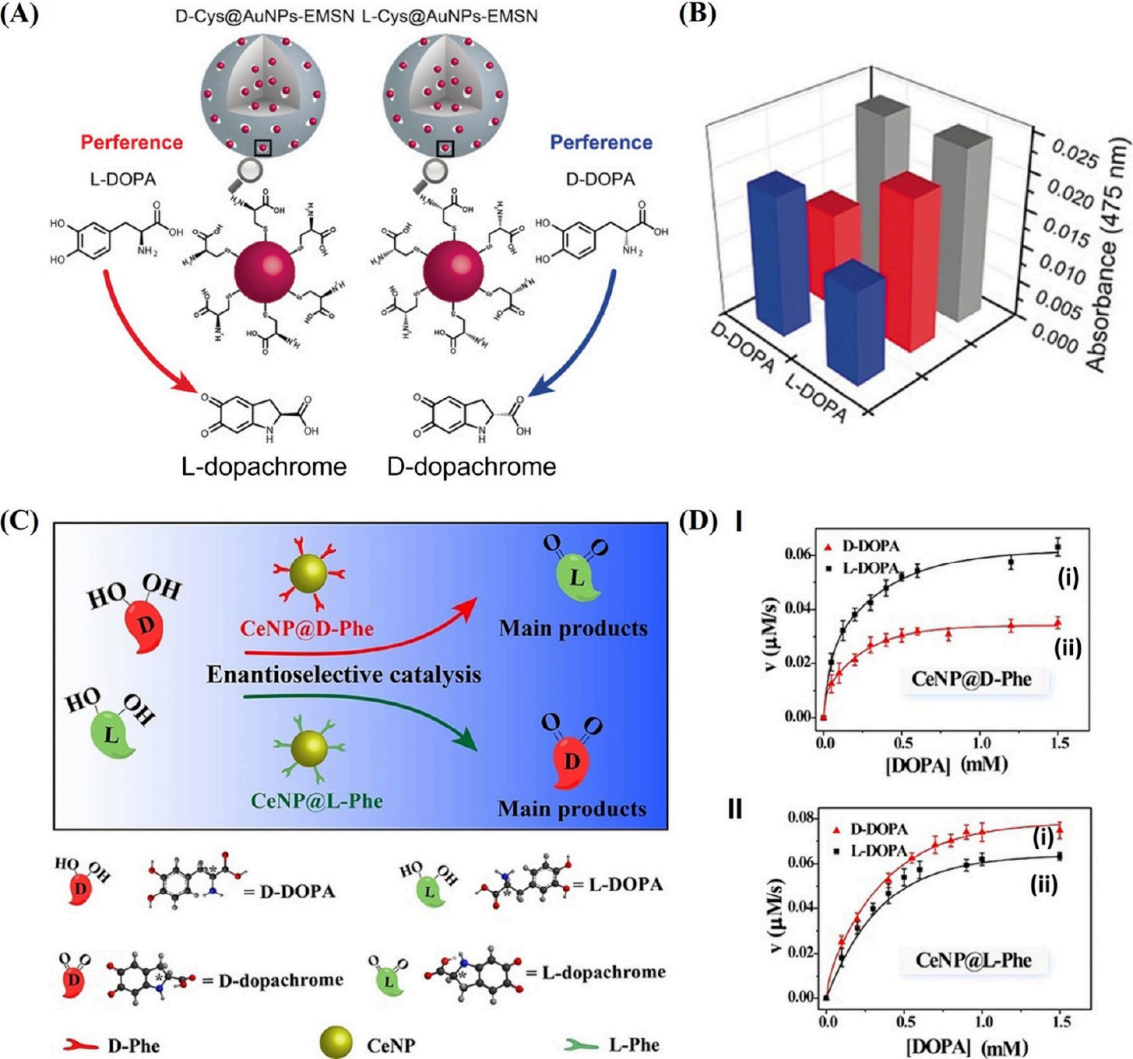
(A) l-/d-cysteine-modified Au NPs encapsulated in SiO_2_ for the chiroselective oxidation of l-/d-DOPA to dopachrome by H_2_O_2_. (B) Absorbance corresponding to the catalyzed H_2_O_2_ oxidation of l-/d-DOPA to dopachrome by using l-cysteine-modified Au NPs/SiO_2_ (blue), d-cysteine-modified Au NPs/SiO_2_ (red), and bare Au NPs/SiO_2_ (gray). Reproduced with permission from ref 60. Copyright 2018 Wiley. (C) Schematic chiroselective aerobic oxidation of l-/d-DOPA by l-or d-phenylalanine-modified CeO_2_ particles to dopachrome. (D) Panel I: rates of l-DOPA (i) and d-DOPA (ii) oxidation catalyzed by the d-phenylalanine-modified CeO_2_. Panel II: rates of d-DOPA (i) and l-DOPA (ii) oxidation catalyzed by the l-phenylalanine-modified CeO_2_. Reproduced with permission from ref 118. Copyright 2017 Wiley.

In a related study,^118^ ceria NPs (CeO_2_, 4.3 nm) exhibiting oxidase-like activity was transformed into chiral nanozyme by primary coating of the CeO_2_ NPs with poly­(acrylic acid) and subsequently covalent linkage of l- or d-phenylalanine (l-/d-Phe) to the coating layer (Figure 5C). While the d-Phe-CeO_2_ NPs showed a ca. 86% enhanced oxidation of l-DOPA as compared to d-DOPA (Figure 5D, Panel I), the l-Phe-CeO_2_ revealed ca. 13% enhanced oxidation of d-DOPA as compared to l-DOPA to yield the respective d-/l-dopachrome (Figure 5D, Panel II). The detailed kinetic analysis of the chiroselective oxidation of variable concentrations of l-DOPA or d-DOPA in the presence of d-Phe-CeO_2_ or l-Phe-CeO_2_ demonstrated that the d-Phe-CeO_2_ nanozyme exhibited *K*_M_ = 0.915 μM, *k*_cat_= 1.94 × 10^–3^ s^–1^ toward d-DOPA and *K*_M_ = 0.168 μM, *k*_cat_= 3.12 × 10^–3^ s^–1^ toward l-DOPA. In turn, the l-Phe-CeO_2_ showed *K*_M_ = 0.431 μM, *k*_cat_= 4.16 × 10^–3^ s^–1^ toward l-DOPA and *K*_M_ = 0.424 μM, *k*_cat_= 4.63 × 10^–3^ s^–1^ toward d-DOPA. The selectivity factors of the chiral nanozymes demonstrated the chiroselective properties of the particles, and the observed difference in the selectivity factors was attributed to the different loading of the chiral Phe ligands on the particles.

A further chiroselective nanozyme based on Fe_3_O_4_ catalyzing the oxidative dimerization of l-/d-tyrosinol was reported.^61^ The Fe_3_O_4_ nanozyme is a well-established peroxidase mimicking particle, and diverse peroxidase-emulating catalytic transformations were driven by this nanozyme. The Fe_3_O_4_ nanozyme was transformed into a chiroselective nanozyme by coating Fe_3_O_4_ particle with a thin SiO_2_ shell to associate the siloxane methacrylate layer. Chiral amino acid-associated acrylic acid monomer was then polymerized onto the siloxane methacrylate to yield chiral l- or d-amino acid modified interfaces in spatial proximity to the Fe_3_O_4_ nanozyme (Figure 6A). The chiral amino-acid-modified Fe_3_O_4_ particles reveal chiroselective H_2_O_2_-driven oxidative dimerization of l- or d-tyrosinol (Figure 6B). The most effective chiral discrimination was demonstrated using l- or d-tryptophan (l-/d-Trp) as particle modifier. In the presence of d-Trp-modified Fe_3_O_4_, the oxidative dimerization of d-tyrosinol by H_2_O_2_ was ca. 5-fold enhanced as compared to the l-tyrosinol, whereas in the presence of l-Trp-functionalized Fe_3_O_4_ particles, the catalyzed H_2_O_2_-driven dimerization of l-tyrosinol was 4-fold enhanced as compared to the d-tyrosinol (Figure 6B).

**Figure 6:**
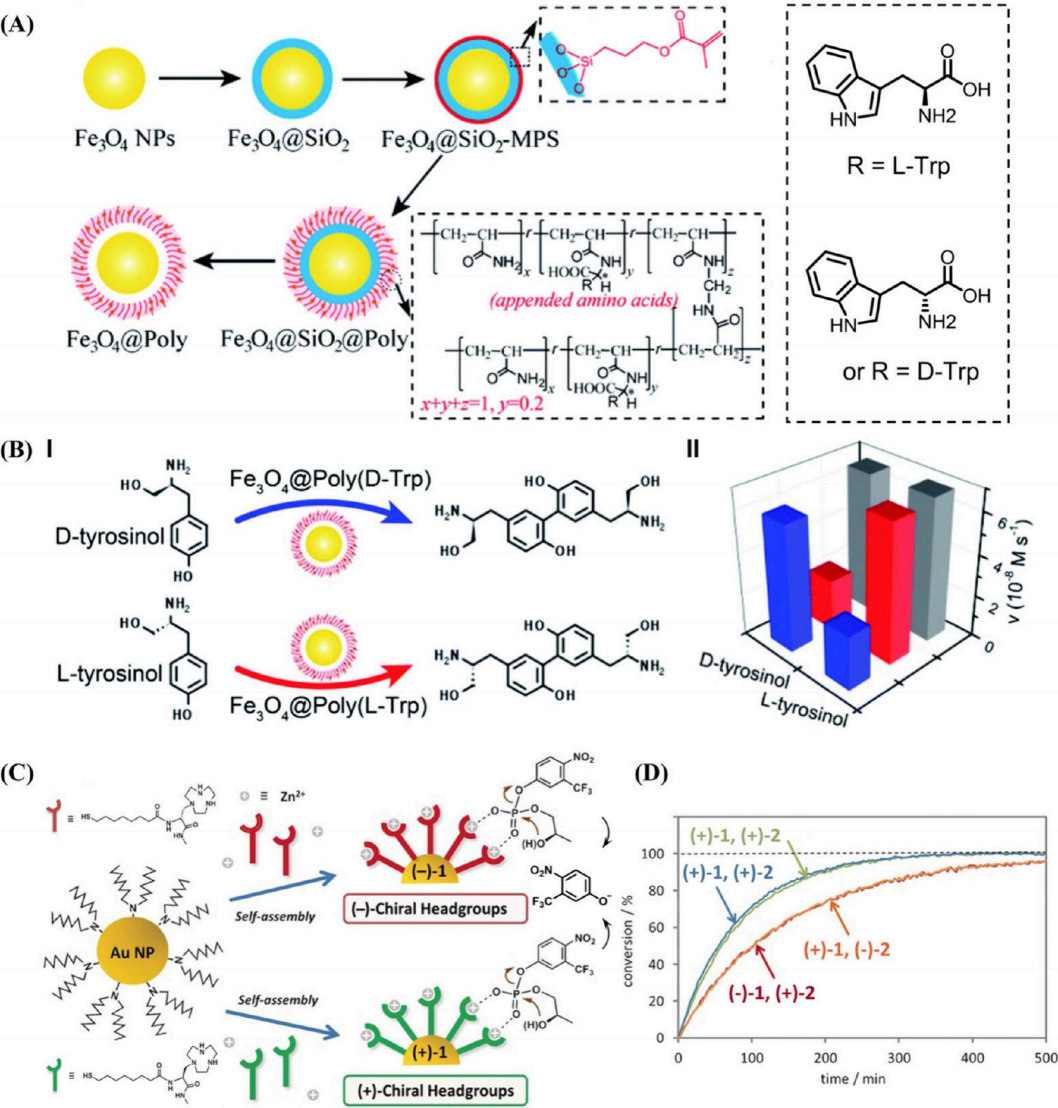
(A) Synthesis of SiO_2_ thin film-assisted l-/d-tryptophan-functionalized polyacrylamide-coated Fe_3_O_4_ NPs as catalyst for the chiroselective coupling of l-/d-tyrosinol. (B) Panel I: schematic chiroselective H_2_O_2_-driven coupling of l-/d-tyrosinol by the chiral l-/d-tryptophan-modified nanoparticles shown in (A). Panel II: rates of l-/d-tyrosinol coupling catalyzed by the d-tryptophan-modified particles (blue), the L-tryptophan-modified particles (red), and the nonmodified particles (gray) in the presence of H_2_O_2_. Reproduced with permission from ref 61. Available under a CC-BY 4.0 license. Copyright 2020 RSC. (C) Schematic chiroselective hydrolysis of (+)/(−)-2-hydroxypropyl p-nitro-m-trifluoromethylphenyl phosphate using Zn^2+^-coordinated (+)/(−)-1-(2-(8-(mercapto)­octanamido)-3-(methylamino)-3-oxopropyl)-1,4,7-triazacyclononane-modified Au NPs as catalysts. (D) Hydrolysis efficacy of Zn^2+^-coordinated (+)/(−)-1-(2-(8-(mercapto)­octanamido)-3-(methylamino)-3-oxopropyl)-1,4,7-triazacyclononane-modified Au NPs toward (+)/(−)-2-hydroxypropyl p-nitro-m-trifluoromethylphenyl phosphate. Reproduced with permission from ref 119. Copyright 2016 Wiley.

A further Au nanoparticle-based nanozyme catalyzing the chiroselective hydrolysis of phosphodiester was developed (Figure 6C).^119^ Au NPs (1.5 nm) were functionalized with (+)/(−)-1-(2-(8-(mercapto)­octanamido)-3-(methylamino)-3-oxopropyl)-1,4,7-triazacyclononane metal ion binding ligands, (+)/(−)-L, and Zn^2+^ was coordinated to the macrocyclic ligand as catalytic units for the chiroselective hydrolysis of the chiral substrate (+)/(−)-2-hydroxypropyl p-nitro-m-trifluoromethylphenyl phosphate, (+)/(−)-**2**. The chiral (+)-l-modified Au NPs, (+)-**1**, revealed enhanced catalytic activity toward the hydrolysis of (+)-**2** to form 4-nitro-3-(trifluoromethyl)­phenol, as compared to the hydrolysis of (−)-**2** (Figure 6D). In turn, the (−)-l-functionalized Au NPs, (−)-**1**, demonstrated opposite chiroselectivity toward hydrolysis of (+)/(−)-**2**. The (−)-l-modified Au NPs presented enhanced hydrolysis of (−)-**2** compared to the hydrolysis of (+)-**2** (Figure 6D). While the present section emphasized the molecular ligand functionalization of the nanozymes as a path to enhance the catalytic activity and selectivity/chiroselectivity of the nanozymes, we note that binding of ions to nanozymes can promote or inhibit the catalytic functions of the nanozymes. For example, acetate ions^120^ associated with Pt nanozyme were reported to promote the peroxidase-like activity of the nanozyme through hydrogen bond-assisted binding of the substrate, whereas halides^121^ or pyrophosphate ions^122^ were reported to inhibit the nanozyme activities through blocking their catalytic sites.

## 4 Functional Polymer-Modified Nanozymes

Molecular imprinted polymer (MIP) matrices represent a broad class of polymers that include specific molecular binding sites generated within the course of synthesis of the polymer frameworks.^123,124^ Two general methods were developed to prepare MIP matrices.^125,126^ By one method, functional monomers interacting with chemical functionalities associated with the molecular ligand by supramolecular interactions, such as electrostatic, H-bond, or donor–acceptor interactions, are polymerized, followed by washing off of the guest ligands, yielding polymer frameworks that include molecular contours exhibiting specific binding for the imprinted guest substrates. By a second approach, the guest substrates are modified with cleavable monomer units. Polymerization of the monomer guest substrates in the presence of the other monomers, under cross-linking conditions, leads, after cleavage of the guest substrate and subsequent washing off of the cleaved substrate, to specific molecular-imprinted polymer matrices. MIP frameworks revealing binding specificity^127^ or chiroselectivity^128^ were prepared by these methods. Diverse applications of MIP frameworks for catalysis,^129,130^ separation,^131,132^ sensing,^133,134^ and controlled release^135,136^ were extensively reviewed. The modification of nanozyme particles with molecularly imprinted polymer coatings provides a versatile means to generate specific substrate binding sites and even chiroselective binding sites in spatial proximity to the nanozyme catalytic sites, thereby enhancing the catalytic performance of nanozymes by concentrating the reaction substrate (“molarity effect”). Moreover, the generation of molecularly imprinted sites in the polymer coating introduces substrate specificity and even chiroselectivity into the nanozyme catalytic features. This approach is not free, however, from limitations as the coating of the nanozyme with polymer might perturb the catalytic sites and introduce permeation barriers for the substrates reaching the catalytic sites. Two general strategies to functionalize nanozymes with MIP layers were reported. By one method, polymerizable monomers were adsorbed onto the nanozyme and the coating monomers were subjected to polymerization in the presence of the reaction substrate to yield the molecular-imprinted coating on the nanozyme particles.^64^ By a second approach, the nanozyme itself acted as catalyst inducing the polymerization process, in the presence of the substrate ligands, to yield the active MIP coating on the nanozyme.^65,137^

Figure 7A depicts the synthesis of peroxidase mimicking nanozyme, Fe_3_O_4_, functionalized with a TMB-imprinted polymer coating or a ABTS^2–^-imprinted polymer coating composed of the mixture of acrylamide, *N*-isopropylacrylamide and N,N′-methylenebis­(acrylamide) monomers.^64^ The MIP matrices were generated by a radical-induced cross-linking polymerization of the monomer constituents, in the presence of TMB or ABTS^2–^ as imprinting substrates. Figure 7B, Panel I depicts the rate of TMB oxidation by H_2_O_2_ in the presence of the TMB-imprinted Fe_3_O_4_ nanozyme, in comparison to the rate of oxidation of TMB by the bare or nonimprinted polymer-coated nanozymes. The TMB-imprinted nanozyme demonstrated a 2.8-fold enhanced oxidation rate of TMB, as compared to the bare nanozyme, consistent with the binding of TMB in the imprinted sites and the local increased concentration of TMB in proximity to the nanozyme catalytic sites (“molarity effect”). In contrast, a slightly lower catalytic activity of the nonimprinted nanozyme, as compared to the bare particle, was observed, suggesting that the coating inhibited the catalytic performance of the nanozyme. Moreover, the ABTS^2–^-imprinted Fe_3_O_4_ nanozyme framework did not show any enhanced activity toward the TMB oxidation implying that the imprinting perforation of the polymer coating results in selective binding sites for the oxidation of TMB. Similar results were observed for the ABTS^2–^-imprinted Fe_3_O_4_ nanozyme (Figure 7B, Panel II). The ABTS^2–^-imprinted nanozyme revealed a 4-fold enhanced peroxidase-like activity toward the oxidation of ABTS to ABTS^–•^ by H_2_O_2_, as compared to the bare particle or the nonimprinted polymer-coated nanozyme, indicating the concentration effect and enhanced oxidation rate by the imprinted framework. Also, the TMB-imprinted nanozyme has no promoting effect on the oxidation rate of ABTS^2–^, demonstrating the selectivity of peroxidase-like activity guided by the imprinting process.

**Figure 7:**
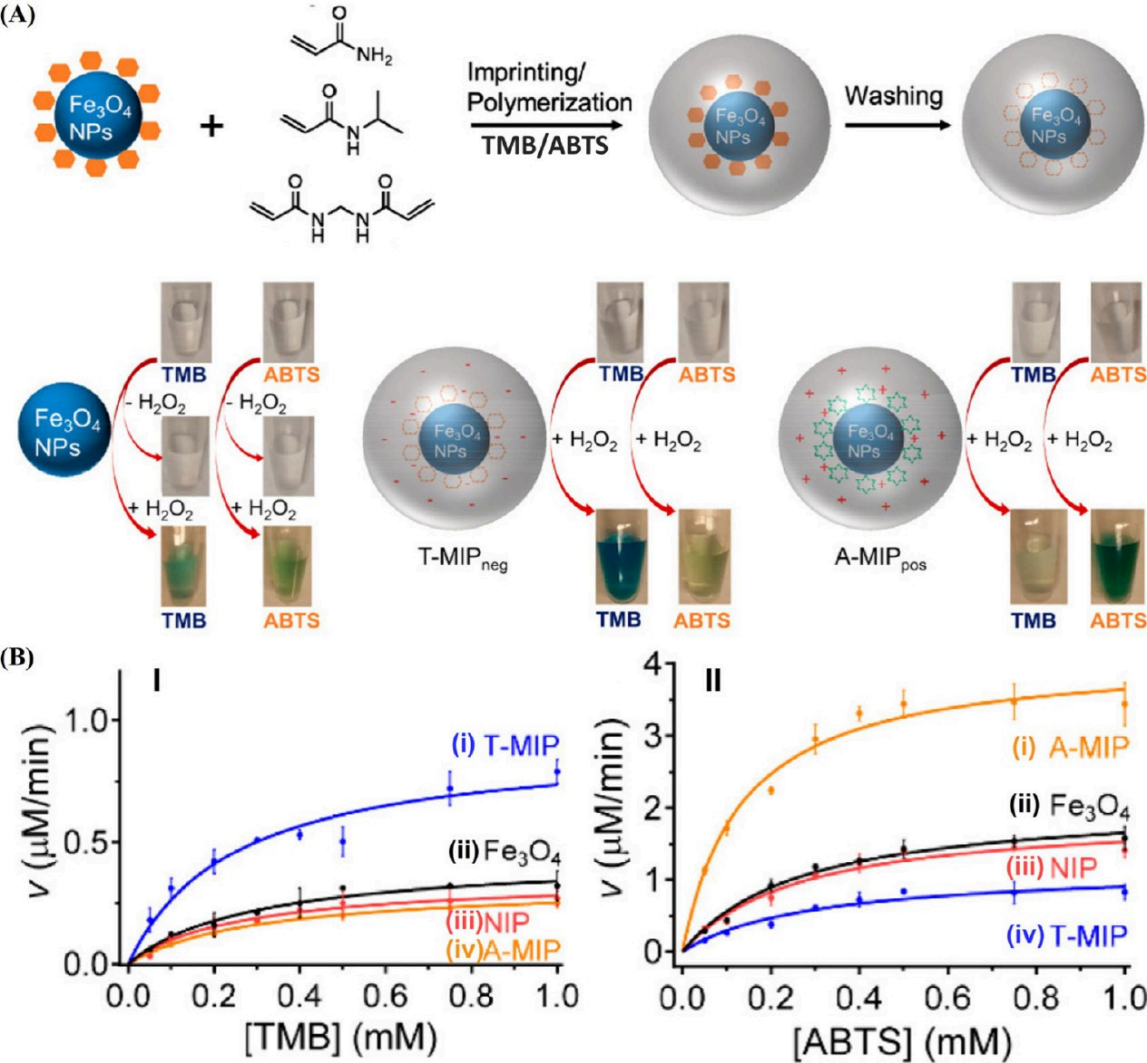
(A) Schematic synthesis of Fe_3_O_4_ particles coated with a TMB or ABTS^2–^-imprinted polyacrylamide/poly­(*N*-isopropylacrylamide) coating for the enhanced and selective oxidation of TMB or ABTS^2–^ by H_2_O_2_. (B) Panel I: rates of TMB oxidation by H_2_O_2_ yielding TMB^+•^ using (i) TMB-imprinted polymer-coated Fe_3_O_4_, (ii) bare Fe_3_O_4_ particles, (iii) nonimprinted polymer-coated Fe_3_O_4_, and (iv) ABTS^2–^-imprinted polymer-coated Fe_3_O_4_. Panel II: rates of ABTS^2–^ oxidation by H_2_O_2_ yielding ABTS^–•^ using (i) ABTS^2–^-imprinted polymer-coated Fe_3_O_4_, (ii) bare Fe_3_O_4_ particles, (iii) nonimprinted polymer-coated Fe_3_O_4_, and (iv) TMB-imprinted polymer-coated Fe_3_O_4_. Reproduced from ref 64. Copyright 2017 ACS.

Nanozymes, such as Co^2+^-ZIF-67 particles, were found to exhibit peroxidase-like activities, such as the catalyzed H_2_O_2_ oxidation of dopamine to aminochrome or oxidation of NADH to NAD^+^. The nanozyme was also found to catalyze the oxidation of aniline to polyaniline, PAn, in the presence of poly­(styrenesulfonate).^65^ The formation of PAn in solution was accompanied by the coating of the ZIF particle with a PAn layer. Accordingly, the nanozyme-catalyzed formation of PAn coating in the presence of nanozyme substrates leads to the entrapment of the substrates in the polymer coating and the subsequent washing off of the bound substrates yielding an imprinted PAn-coating nanozyme framework that includes selective or chiroselective binding sites for the imprint substrate molecular (polynanozyme). Figure 8A depicts the schematic formation of the PAn-coated Co^2+^-ZIF-67 particles and their structural and compositional features. The Co^2+^-ZIF-67 particles were generated by the reaction between Co^2+^ and 2-methylimidazole, and the resulting crystalline framework exhibited a cubic crystalline structure (Panel I). The resulting Co^2+^-ZIF-67 particles catalyzed the H_2_O_2_ oxidation of aniline to PAn (Panel II). The coating of the particles with the polymer layer was visualized by SEM (Panel III) and element mapping (Panel IV). The Co^2+^-ZIF-67 nanozyme was then applied to catalyze the assembly of chiroselective l-/d-DOPA-imprinted PAn coating on the Co^2+^-ZIF-67 nanozyme, polynanozyme (Figure 8B). The coating of the nanozyme with the l-/d-DOPA-imprinted sites involved the nanozyme-catalyzed oxidation of aniline by H_2_O_2_ in the presence of l- or d-DOPA and the subsequent washing off of the imprinting substrate to yield chiroselective l-/d-DOPA-imprinted PAn-coated nanozyme frameworks, catalyzing the nanozyme-driven chiroselective oxidation of l- or d-DOPA by H_2_O_2_ to form dopachrome. The chiroselective catalytic features of the l-/d-DOPA-imprinted Co^2+^-ZIF-67 nanozyme are displayed in Figure 8C. While the l-DOPA-imprinted PAn-coated nanozyme revealed superior oxidation of l-DOPA as compared to d-DOPA by H_2_O_2_ to form dopachrome (Panel I), reversed chiroselective oxidation of l-/d-DOPA was demonstrated by the d-DOPA-imprinted PAn-coated nanozyme. The oxidation rate of d-DOPA by H_2_O_2_ to dopachrome by the d-DOPA-imprinted PAn-coated nanozyme was faster as compared to the oxidation of l-DOPA to dopachrome (Panel II). Noteworthy is the fact that the nonimprinted PAn-coated Co^2+^-ZIF-67 revealed a substantially lower catalytic activity toward the oxidation of l-/d-DOPA. Thus, the imprinting of the polymer coating with chiral recognition sites not only induces chiroselective oxidation of l-/d-DOPA but also provides sites for concentrating the substrates in spatial proximity to the catalytic sites (“molarity effect”). The nonimprinted PAn coating blocks the permeation of the l-/d-DOPA reaction substrates to the catalyst interfaces, leading to the low activity of the nanozyme.

**Figure 8:**
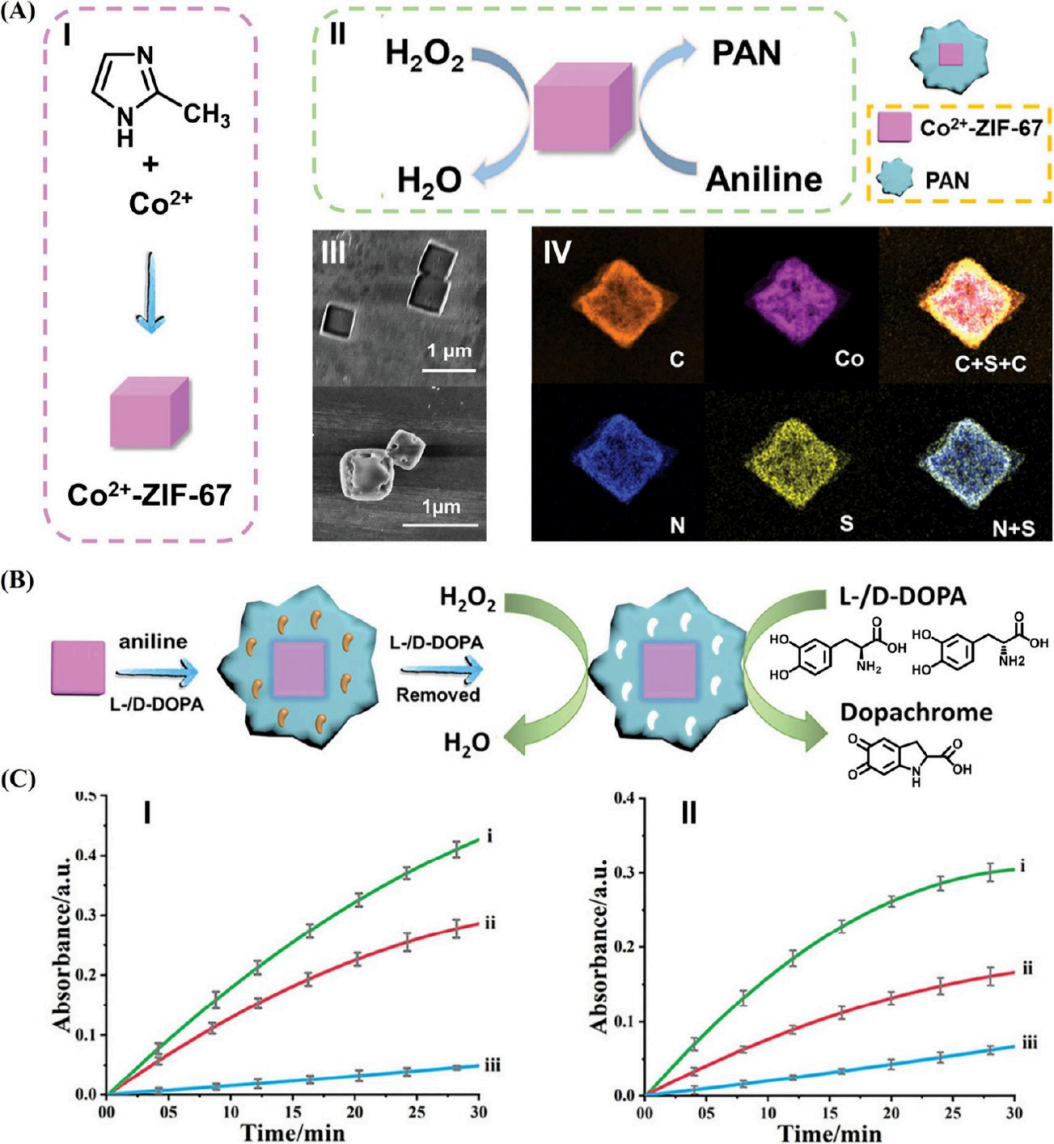
(A) Panel I: schematic synthesis of Co^2+^-ZIF-67 NPs. Panel II: Co^2+^-ZIF-67-catalyzed oxidation of aniline to polyaniline (PAn) by H_2_O_2_ yielding PAn-coated Co^2+^-ZIF-67. Panel III: SEM images corresponding to bare Co^2+^-ZIF-67 particles (upper image) and PAn-coated Co^2+^-ZIF-67 particles (lower image). Panel IV: element mapping of the PAn-coated Co^2+^-ZIF-67 NPs. (B) Schematic imprinting of l-/d-DOPA sites into PAn-coated Co^2+^-ZIF-67 NPs for the chiroselective and enhanced H_2_O_2_ oxidation of l-/d-DOPA. (C) Panel I: temporal absorbance changes corresponding to (i) oxidation of l-DOPA in the presence of l-DOPA-imprinted NPs and H_2_O_2_, (ii) oxidation of d-DOPA in the presence of l-DOPA-imprinted NPs and H_2_O_2_, and (iii) oxidation of l-DOPA in the presence of nonimprinted PAn-coated NPs and H_2_O_2_. Panel II: temporal absorbance changes corresponding to (i) oxidation of d-DOPA in the presence d-DOPA-imprinted NPs and H_2_O_2_; (ii) oxidation of l-DOPA in the presence d-DOPA-imprinted NPs and H_2_O_2_; (iii) oxidation of d-DOPA in the presence nonimprinted PAn-coated NPs and H_2_O_2_. Reproduced from ref 65. Available under a CC-BY 4.0 license. Copyright 2023 Wiley.

Another molecular-imprinted PAn-coated nanozyme included uric acid (UA)-imprinted Cu-ZIF nanoparticles catalyzing the aerobic oxidation of UA to allantoin, mimicking the function of native uricase.^137^ The oxidation of UA has therapeutic significance since deposition of UA crystals in peripheral joints and tissues causes the inflammatory arthritis gout.^115^ As uricase catalyzes the degradation of UA crystals in nature,^138^ substantial efforts have been directed toward the development of synthetic agents substituting uricase deficiency, and beyond, monitoring and sensing UA levels in body fluids.^116,117^ Cu-ZIF^137^ synthesized by reacting Cu^2+^ with 2-methylimidazole demonstrated peroxidase-like activity toward the oxidation of UA to allantoin by H_2_O_2_ (Figure 9A, Panel I). In addition, the Cu-ZIF revealed catalytic activities toward the oxidation of aniline to PAn, in the presence of H_2_O_2_, resulting in the coating of the Cu-ZIF particle with a PAn layer (Panel II). Accordingly, imprinting of UA sites in the PAn coating of Cu-ZIF particles was examined as a means to synthesize and improve nanozyme for the oxidation of UA. In these experiments, the Cu-ZIF-catalyzed oxidation of aniline to PAn, in the presence of UA, was conducted. The electrostatic and H-bond interactions between the PAn film and UA were envisaged to generate affinity interactions between the UA guest ligands and the PAn polymer coating. After the bound UA units were washed off, a UA-imprinted PAn coating on the nanozyme was formed (Figure 9B, Panel I). Figure 9, Panel II presents the concentration changes of UA upon H_2_O_2_-driven oxidation in the presence of a bare Cu-ZIF framework, UA-imprinted polynanozyme, and the nonimprinted PAn-coated Cu-ZIF. While the catalyzed H_2_O_2_-driven oxidation of UA by the UA-imprinted polynanozyme was substantially enhanced as compared to the bare Cu-ZIF nanozyme, the oxidation of UA by the nonimprinted Cu-ZIF nanozyme was significantly inhibited as compared to the bare Cu-ZIF nanozyme. These results were consistent with the fact that the UA-imprinted coating provides a functional interface to bind the UA substrates in spatial proximity to the catalytic nanozyme (“molarity effect”). In turn, the inhibitory effect on the oxidation of UA by the nonimprinted PAn-coated nanozyme, as compared to the bare particle, was attributed to the polymer-induced perturbation of the accessibility of UA to the catalytic sites by blockage of the substrate permeability through the nonimprinted PAn coating.

**Figure 9:**
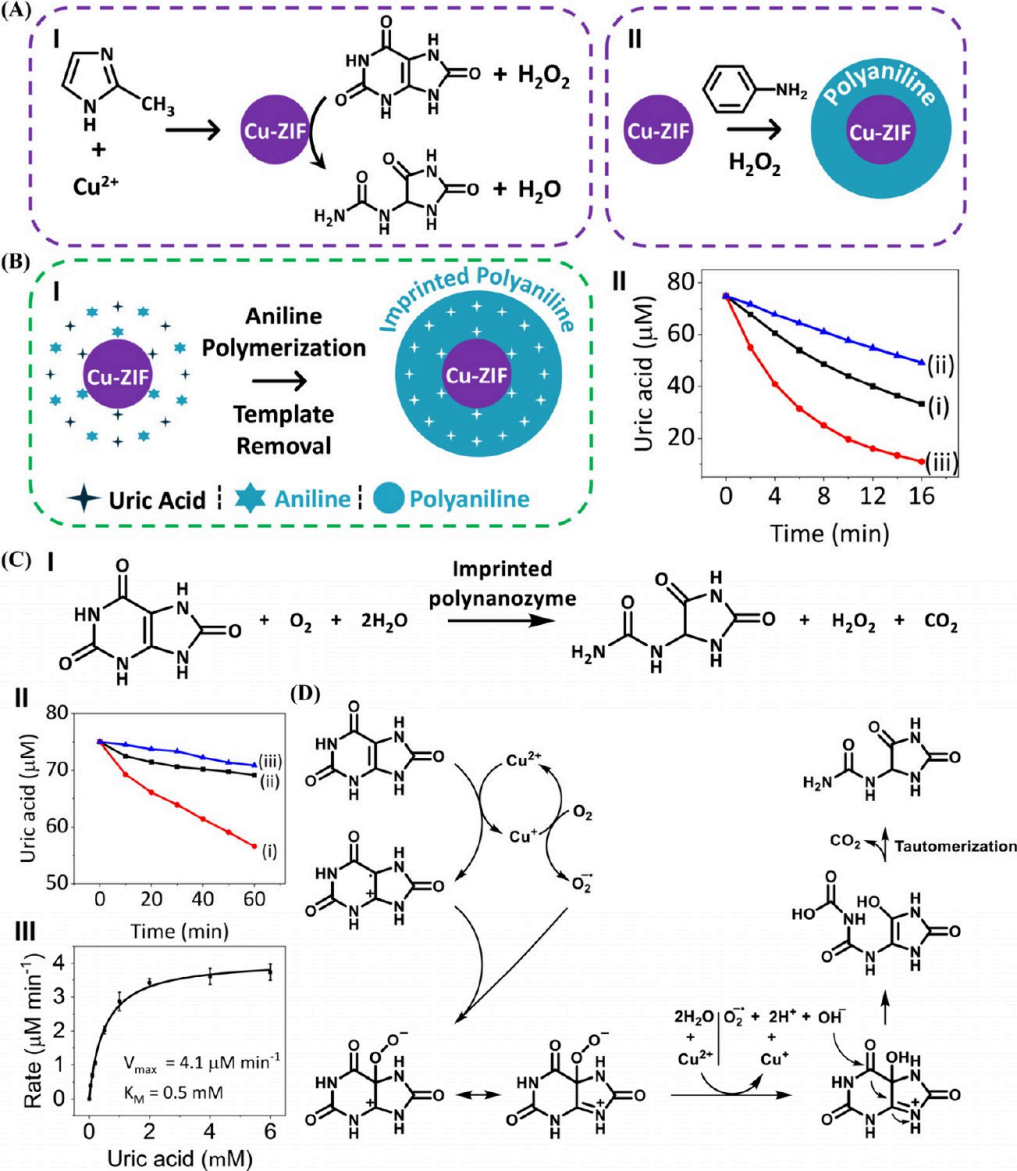
(A) Panel I: schematic synthesis of Cu-ZIF NPs for the catalyzed H_2_O_2_ oxidation of uric acid (UA) to allantoin. Panel II: Cu-ZIF-catalyzed oxidation of aniline to polyaniline (PAn) by H_2_O_2_ yielding PAn-coated Cu-ZIF NPs. (B) Panel I: schematic imprinting of UA sites in PAn-coated Cu-ZIF nanoparticles. Panel II: temporal concentration changes of UA upon the H_2_O_2_-driven oxidation of UA to allantoin in the presence of (i) bare Cu-ZIF particles, (ii) nonimprinted PAn-coated Cu-ZIF particles, and (iii) UA-imprinted PAn-coated Cu-ZIF particles. (C) Panel I: schematic aerobic oxidation of UA to allantoin by the UA-imprinted Cu-ZIF particles. Panel II: temporal concentration changes of UA upon aerobic oxidation to allantoin in the presence of (i) UA-imprinted Cu-ZIF particles, (ii) bare Cu-ZIF particles, and (iii) nonimprinted PAn-coated Cu-ZIF particles. Panel III: kinetic analysis of the aerobic oxidation of UA by UA-imprinted PAn-coated Cu-ZIF polynanozyme. (D) Suggested mechanism for the aerobic oxidation of UA to allantoin in the presence of the UA-imprinted PAn-coated Cu-ZIF polynanozyme. Reproduced from ref 137. Available under a CC-BY 4.0 license. Copyright 2025 ACS.

However, the UA-imprinted PAn-coated Cu-ZIF nanozyme also exhibited oxidase-like activity, reflected by the aerobic oxidation of UA to allantoin (Figure 9C, Panel I). The imprinted polynanozyme demonstrated effective aerobic oxidation of UA to allantoin, *V*_max_ = 4.1 μM min^–1^, *K*_M_ = 0.5 mM, while the bare Cu-ZIF particle or the nonimprinted PAn-coated Cu-ZIF lacked oxidase-like activities (Panels II and III). The discovery of the oxidase-like catalytic activities of the UA-imprinted PAn-coated nanozyme, a catalytic feature lacking in the bare particle, is unique, as it demonstrates the emergence of a new catalytic hybrid framework originating from the polymer imprinting process. Mechanistic studies attempting to elucidate the origin of the oxidase-like activity of the polynanozyme revealed that within the process of imprinting the PAn coating with the UA sites, the Cu^2+^ constituent in Cu-ZIF nanozyme is partially reduced to Cu^+^ and the generated Cu^+^ species participated, then, in the reduction of O_2_ to O_2_^–•^. The intermediate formation of O_2_^–•^ and UA^+•^ provided the basic insight into formulating a mechanistic pathway for the aerobic oxidation of UA to allantoin (Figure 9D).

Beyond enhanced/selective catalytic/binding properties of imprinted polymer-coated nanozymes, tailored polymer coating associated with nanozymes demonstrated targeted antibacterial cytotoxicity or dictated anti-inflammatory functions (Figure 10). This is exemplified by the synthesis of benzeneboronic acid-functionalized octadecyl (C_18_)-polyethylene glycol (CPB) (Figure 10A, Panel I).^139^ The CPB polymer that can bind peptidoglycan on the bacterial cell wall was used to coat graphene-encapsulated PtCo nanocrystals (PtCo@G), exhibiting oxidase-like activities catalyzing the generation of a superoxide radical (Panel II). Subjecting the CPB-functionalized PtCo@G (PtCo@G@CPB) nanozyme to (), resulted in the selective and efficient ROS-induced killing of the bacteria (Panel III). Also, Figure 10B depicts the application of tannic acid (TA)-stabilized Ce^3+/4+^ (CeTA) as nanozymes for the effective depletion of ROS associated with viral pneumonia.^140^ This is exemplified with the functionalization of the CeTA nanozyme with a thioketal-bridged polypeptide and poly­(ethylene glycol) (K_1_tkP), (Figure 10B, Panel I). The polymer-coated CeTA releases polyethylene glycol through the cleavage of thioketal linker upon high ROS stimulation and subsequently self-assembles into fibrous CeTA (CeTA-*k*_1_tkP) due to the peptide aggregation into the β-sheet, which retains long-term intracellular ROS depletion efficacy. Effective control over the inflammatory cytokines (IL-6; TNF-α; IL-1β) associated with viral pneumonia through the CeTA-*k*_1_tkP nanozyme were demonstrated (Panel II).

**Figure 10:**
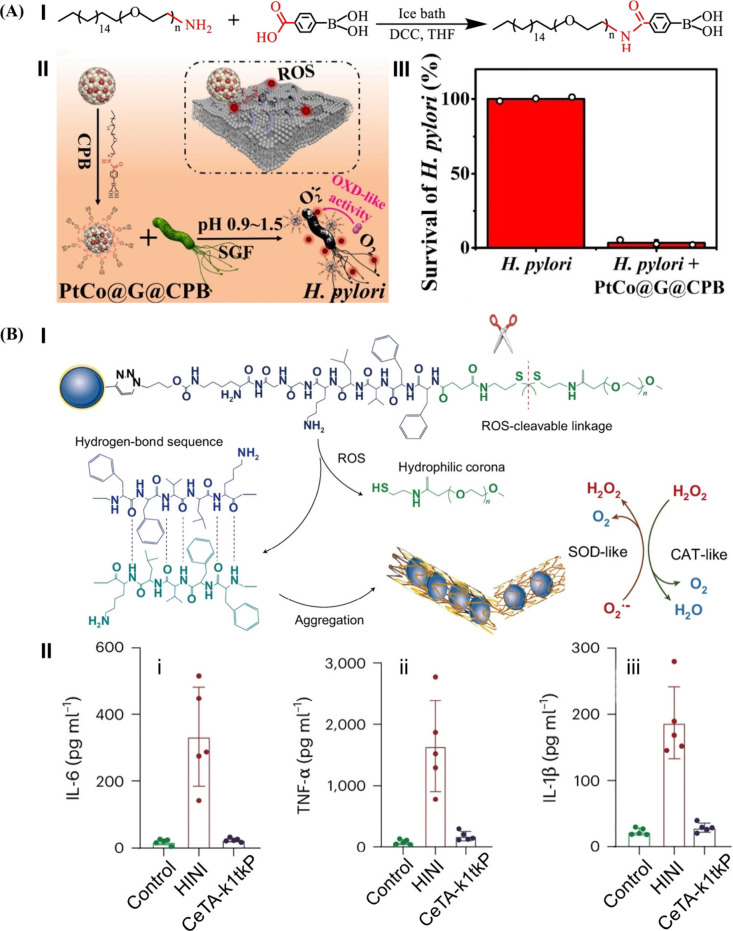
(A) Panel I: schematic synthesis of benzeneboronic acid-functionalized octadecyl (C18)-polyethylene glycol (CPB). Panel II: schematic synthesis of CPB-modified graphene-encapsulated PtCo nanocrystals (PtCo@G@CPB) and antibacterial mechanism of PtCo@G@CPB against . Panel III: survival of treated with PtCo@G@CPB. Reproduced from ref 139. Available under a CC-BY 4.0 license. Copyright 2025 Springer Nature. (B) Panel I: schematic illustration of the self-assembly and the enzyme-like activities of the CeTA-K_1_tkP nanozyme. Panel II: production of inflammatory cytokines, IL-6 (i), TNF-α (ii), and IL-1β (iii), in bronchoalveolar lavage fluid of Flu A virus (H1N1)-mediated pneumonia model in mice after treatment by CeTA-K_1_tkP nanozyme as compared to the no treatment system. Reproduced with permission from ref 140. Copyright 2025 Spring Nature.

## 5 Surface-Modified Nanozymes for Sensor Applications

This review has emphasized surface modification strategies for the enhanced catalytic performance of nanozymes. These included the modification of the nanozymes with aptamers, synthetic molecular or macromolecular receptors, and functional polymer coatings. Three complementary functions demonstrated by the surface-modified nanozyme hybrids in enhancing the catalytic performance of the systems were emphasized. These involved the binding of the reaction substrates by the modifying interface in spatial proximity to the catalytic sites, leading to *a “molarity effect”*, *high turnover of the catalytic process* within the confined microenvironment, and *high selectivity/chiroselectivity* of the reactions. These features of the surface-modified nanozymes are key parameters of sensing platforms, where analytes are concentrated by the recognition interfaces, leading to the selectivity and amplification of the recognition events that allow the sensitive sensing of the target analyte. Indeed, surface-modified nanozymes have found broad interest for the development of optical or electrochemical sensors and comprehensive review articles have addressed the topic.^69,141^ The different strategies of surface modification of nanozymes were accompanied by specific examples of possible sensing application, e.g., dopamine, uric acid, and glucose. We feel, however, that it is important to compare the capacities of the different strategies for sensing, addressing the advantages, limitations, and future challenges of applying the surface-modified nanozymes for sensing. This comparison is provided in Table 1.

**Table 1:** Advantages, Limitations, and Future Challenges of Surface-Modified Nanozymes for Sensing

Surface Modification Strategy	Advantages	Limitations	Future Challenges
Aptamer-modified nanozyme	• High and selective binding affinities	• Sensitivity toward enzymatic/bacterial degradation	• Expand aptamer availability
	• Possibility to use aptamer libraries	• Operation at restricted environmental conditions (pH, ionic strength, temperature)	• Improve aptamer binding capacities by structural base mutations^142,143^
	• Stimuli-responsive and switchable binding sites	• Cost effectiveness and scalability	• Stimuli-responsive aptamers
		• Limited availability of aptamers toward low-molecular-weight ligands	• Apply AI or machine learning technologies for prediction of aptamer binding capacities
			• Identify aptamer-based nanozymes for theranostic applications
			
Receptor-modified nanozyme	• Synthetic processability	• Low or moderate binding affinities due to limited motives	• Improve binding capacities, selectivity and chiroselectivity by chemical modification of the receptors
	• Scalability and cost effectiveness	• Limited availability of synthetic receptors	• Develop new synthetic methods to anchor receptors to nanozyme interfaces
	• Relative long-term stability		• Apply innovative nanozyme-driven transduction signals, e.g., SERS^144^
			• Apply modified nanozyme for nonconvention substrate samples^145^
			
Polymer-modified nanozyme	• Easy design of molecular-imprinted binding sites	• Low or moderate binding affinities	• Design stimuli-responsive (light or redox) imprinted polymers
	• Adaptation to diverse substrates	• Low density of specific binding sites	• Implement nanozymes as catalysts to generate imprinted polymer coatings
	• High stability	• Permeability barrier for binding guest substrates	

## 6 Conclusions and Perspectives

The review article introduced different methods to enhance the catalytic activities of nanozymes and improve their selective and chiroselective properties through surface modification of the nanozyme frameworks. These included the functionalization of the nanozymes with sequence-specific aptamer binding strands, the modification of the nanozymes with substrate-specific binding receptors, such as cyclodextrins or affinity ligands, e.g., amino acids or peptides, and the coating nanozyme frameworks with molecular-imprinted polymer matrices binding the reaction substrates. (For a comprehensive comparison of the catalytic performance and selectivities of the surface-modified nanozyme systems discussed in the article, see Table S1 in the Supporting Information.) The binding and concentration of the reaction substrates to the surface modifiers, in spatial proximity to the catalytic sites of the nanozymes, were the key motif to emulate native enzyme functions by concentrating the substrate (“molarity effect”) and eventually chiroselective structural alignment of the substrates in proximity to the catalytic sites. Nevertheless, it is well recognized that the high catalytic turnover features of the native enzyme emerge from a hierarchical layer environment allowing the sequential, programmed activation of the substrate in the active site environment. Emulating these native features by hierarchically programed synthetic ligand/polymer layers or single atom modification of the nanozymes are promising approaches to reach these goals.^146,147^ Diverse applications of the surface-modified hybrid structures were demonstrated for sensitive and selective sensing and medical therapeutic uses. Besides, the advances demonstrated by surface-modified nanozymes, future development using these hybrid frameworks, can be envisaged. While the review article has emphasized the surface modification of the nanozymes as a means to promote the catalytic and selectivity functions of the nanozymes, many of the nanozyme frameworks, particularly the metal–organic framework, exhibit porous structures. This calls for the possible modification of the pores with molecular, biomolecular, or polymer agents to modulate the functions of the nanozymes. Indeed, a recent study reported the modulation of nanozyme functions through modification of the pore framework.^148^ Furthermore, most of the nanozymes demonstrated oxidoreductase-like catalytic functions; expanding the scope of nanozymes and their surface modification to other chemical transformations is an important path to follow. Also, functionalization of the nanozymes with stimuli-responsive modifiers, e.g., redox-responsive^149^ or photoresponsive^150^ aptamers, ligands, or molecular-imprinted polymer coatings, is anticipated to yield switchable nanozymes with controllable catalytic activities. Moreover, the synthesis of chiral nanoparticles has significantly advanced in recent years.^151,152^ Surface modification of the chiral nanoparticles with surface modifiers is, in principle, anticipated to yield effective chiral nanozymes. Particularly, functionalization of chiral nanozyme frameworks with chiral modifying ligands is envisaged as a powerful means to generate a plethora of diastereomeric sets of nanozyme/modifier hybrids for diverse selective chemical transformations. Finally, diverse nanoparticles, particularly semiconductor nanoparticles, demonstrated photocatalytic properties.^153–155^ Surface modification of these particles is anticipated to establish rich “photonanozyme” frameworks for different applications.

## Supplementary Material



## Notes

Table S1 describing the comparison of the catalytic performance and selectivity of the surface-modified nanozymes vs the bare nanozymes discussed in the review (PDF)
