# Supplementary material for: Surface-Modified Nanozymes for Enhanced and Selective Catalysis
Source: ACS Appl Mater Interfaces. 2025 Jul 23;17(31):44011–29. doi: 10.1021/acsami.5c07647 (PMC12332837; doi:10.1021/acsami.5c07647)
Supplement: Supplementary file 1 [file am5c07647_si_001.pdf]

Supporting Information for

# Surface-Modified Nanozymes for Enhanced and Selective Catalysis

Xinghua Chen and Itamar Willner*

Institute of Chemistry, The Hebrew University of Jerusalem, Jerusalem 91904, Israel

*E-mail: itamar.willner@mail.huji.ac.il

**Tabel S1.** Comparison of the catalytic performance and selectivity of the surface-modified nanozymes vs. the bare nanozymes discussed in the review article.

| Materials | Enzyme Mimic | Modifiers | Substrates | Enhanced Catalytic Activity[a] | Enhanced Substrate Selectivity | Ref. |
|---|---|---|---|---|---|---|
| $Cu^{2+}$-modified carbon dots | Peroxidase | Dopamine aptamer | Dopamine/$H_2O_2$ | 50-fold | – | 1 |
| | | | L-/D-DOPA/$H_2O_2$ | – | 2-fold | |
| | | L-Tyrosinamide aptamer | L-tyrosinamide/$H_2O_2$ | 15-fold | | |
| $Ce^{4+}$-modified carbon dots | Peroxidase | Dopamine aptamer | Dopamine/$H_2O_2$ | 14-fold | – | 2 |
| Au NPs | Glucose oxidase | Single-strand DNA | L-/D-glucose/$O_2$ | – | 1.3-fold | 3 |
| | | Structure DNA | D-/L-glucose/$O_2$ | – | 1.4-fold | |
| $Fe_3O_4$ | Hydroxylase | Tyrosine aptamer | Tyrosine/AA/$H_2O_2$ | 1.4-fold | – | 4 |
| $Cu^{2+}$-modified carbon dots | Peroxidase | β-cyclodextrin | Dopamine/$H_2O_2$ | 4-fold | – | 5 |
| Pd@Au | Peroxidase | β-cyclodextrin | TMB/$H_2O_2$ | 18-fold | – | 6 |
| Rh/rGO | Uricase | Oligopeptide | Uric acid/$O_2$ | 42-fold | – | 7 |
| $SiO_2$-immobilized Au | Peroxidase | L-cysteine | D-/L-DOPA/$H_2O_2$ | – | 1.7-fold | 8 |
| | | D-cysteine | L-/D-DOPA/$H_2O_2$ | – | 1.5-fold | |

**Table S1.** (Continued)

| | | | | | | |
|---|---|---|---|---|---|---|
| $CeO_2$ | Oxidase | L-phenylalanine | D-/L-DOPA/$H_2O_2$ | – | 1.1-fold | 9 |
| | | D-phenylalanine | L-/D-DOPA/$H_2O_2$ | – | 1.9-fold | |
| $Fe_3O_4$ | Peroxidase | L-tryptophan | L-/D-tyrosinol/$H_2O_2$ | – | 4-fold | 10 |
| | | D-tryptophan | D-/L-tyrosinol/$H_2O_2$ | – | 5-fold | |
| $Zn^{2+}$/trazacyclononane/Au | Hydrolase | (+)-ligand | (+)/(-)-2-hydroxypropyl p-nitro-m-trifluoromethylphenyl phosphate | – | 1.2-fold | 11 |
| | | (-)-ligand | (-)/(+)-2-hydroxypropyl p-nitro-m-trifluoromethylphenyl phosphate | – | 1.2-fold | |
| $CoFe_2O_4$ | Peroxidase | glutamic acid | TMB/$H_2O_2$ | 1.2-fold | – | 12 |
| $Fe_3O_4$ | Peroxidase | TMB-imprinted polymer | TMB/$H_2O_2$ | 2.8-fold | – | 13 |
| | | | TMB/$ABTS^{2-}$/$H_2O_2$ | – | 1.8-fold | |
| | | $ABTS^{2-}$-imprinted polymer | $ABTS^{2-}$/$H_2O_2$ | 4-fold | – | |
| | | | $ABTS^{2-}$/TMB/$H_2O_2$ | – | 32-fold | |
| $Co^{2+}$-ZIF-67 | Peroxidase | L-DOPA-imprinted polymer | L-DOPA/$H_2O_2$ | 8-fold | – | 14 |
| | | | L-/D-DOPA/$H_2O_2$ | – | 1.5-fold | |
| | | D-DOPA-imprinted polymer | D-DOPA/$H_2O_2$ | 6-fold | – | |
| | | | D-/L-DOPA/$H_2O_2$ | – | 2-fold | |
| Cu-ZIF | Peroxidase | Uric acid-imprinted polymer | Uric acid/$H_2O_2$ | 6-fold | – | 15 |

**Table S1.** (Continued)

| | | | | | | |
|---|---|---|---|---|---|---|
| Au NPs | Glucose Oxidase | Glucose- imprinted polymer | Glucose/$O_2$ | 113-fold | – | [16] |

a. Catalytic activity of the surface-modified nanozymes vs. bare nanozymes

Reference


1 Ouyang, Y.; Biniuri, Y.; Fadeev, M.; Zhang, P.; Carmieli, R.; Vázquez-González, M.; Willner, I., Aptamer-modified $Cu^{2+}$-functionalized C-dots: Versatile means to improve nanozyme activities-“aptananozymes”. *J. Am. Chem. Soc.* **2021,** *143* (30), 11510-11519.

2 Ouyang, Y.; Fadeev, M.; Zhang, P.; Carmieli, R.; Sohn, Y. S.; Karmi, O.; Qin, Y.; Chen, X.; Nechushtai, R.; Willner, I., Aptamer-functionalized $Ce^{4+}$-ion-modified C-dots: Peroxidase mimicking aptananozymes for the oxidation of dopamine and cytotoxic effects toward cancer cells. *ACS Appl. Mater. Interfaces* **2022,** *14* (50), 55365-55375.

3 Zhan, P.; Wang, Z.-G.; Li, N.; Ding, B., Engineering gold nanoparticles with DNA ligands for selective catalytic oxidation of chiral substrates. *ACS Catal.* **2015,** *5* (3), 1489-1498.

4 Fang, X.; Yuan, M.; Zhao, F.; Yu, A.; Lin, Q.; Li, S.; Li, H.; Wang, X.; Yu, Y.; Wang, X.; Lin, Q.; Lu, C.; Yang, H., In situ continuous Dopa supply by responsive artificial enzyme for the treatment of Parkinson’s disease. *Nat. Commun.* **2023,** *14* (1), 2661.

5 Wang, S.; Cazelles, R.; Liao, W.-C.; Vázquez-González, M.; Zoabi, A.; Abu-Reziq, R.; Willner, I., Mimicking horseradish peroxidase and NADH peroxidase by heterogeneous $Cu^{2+}$-modified graphene oxide nanoparticles. *Nano Lett.* **2017,** *17* (3), 2043-2048.

6 Li, F.; Hu, Y.; Zhao, A.; Xi, Y.; Li, Z.; He, J., β-cyclodextrin coated porous Pd@Au nanostructures with enhanced peroxidase-like activity for colorimetric and paper-based determination of glucose. *Microchim. Acta* **2020,** *187* (8), 425.

7 Liu, Y.; Li, N.; Su, K.; Du, J.; Guo, R., Arginine-rich peptide–rhodium nanocluster@reduced graphene oxide composite as a highly selective and active uricase-like nanozyme for the degradation of uric acid and inhibition of urate crystal. *Inorg. Chem.* **2024,** *63* (29), 13602-13612.

8 Zhou, Y.; Sun, H.; Xu, H.; Matysiak, S.; Ren, J.; Qu, X., Mesoporous encapsulated chiral nanogold for use in enantioselective reactions. *Angew. Chem. Int. Ed.* **2018,** *130* (51), 17033-17037.

9 Sun, Y.; Zhao, C.; Gao, N.; Ren, J.; Qu, X., Stereoselective nanozyme based on ceria nanoparticles engineered with amino acids. *Chem. Eur. J.* **2017,** *23* (72), 18146-18150.

10 Zhou, Y.; Wei, W.; Cui, F.; Yan, Z.; Sun, Y.; Ren, J.; Qu, X., Construction of a chiral artificial enzyme used for enantioselective catalysis in live cells. *Chem. Sci.* **2020,** *11* (41), 11344-11350.

11 Chen, J. L. Y.; Pezzato, C.; Scrimin, P.; Prins, L. J., Chiral nanozymes-gold nanoparticle-based transphosphorylation catalysts capable of enantiomeric discrimination. *Chem. Eur. J.* **2016,** *22* (21), 7028-7032.

12 Bagdeli, S.; Abbasi Kajani, A.; Taheri-Kafrani, A., Bioinspired amino acid-functionalized cobalt ferrite nanocomposite: A nanozyme-based colorimetric sensor for sensitive and selective quantification of phenolic compounds and ascorbic acid antioxidant capacity. *Food Chem.* **2024,** *457* (1), 140144.

13 Zhang, Z.; Zhang, X.; Liu, B.; Liu, J., Molecular imprinting on inorganic nanozymes for hundred-fold enzyme specificity. *J. Am. Chem. Soc.* **2017,** *139* (15), 5412-5419.

14 Wu, Y.; Qin, Y.; Muppidathi, M.; Carmieli, R.; Fadeev, M.; Lei, W.; Xia, M.; Willner, I., Functional nanozymes consisting of $Co^{2+}$-ZIF-67 metal-organic framework nanoparticles and $Co^{2+}$-ZIF-67/polyaniline conjugates. *Adv. Funct. Mater.* **2023,** *34* (3), 2306929.

15 Chen, X.; Wu, Y.; Qin, Y.; Carmieli, R.; Popov, I.; Gutkin, V.; Fan, C.; Willner, I., Molecularly imprinted polyaniline-coated Cu-zeolitic imidazolate framework nanoparticles: Uricase-mimicking “polynanozyme” catalyzing uric acid oxidation. *ACS Nano* **2025,** *19* (10), 9981-9993.

16 Fan, L.; Lou, D.; Wu, H.; Zhang, X.; Zhu, Y.; Gu, N.; Zhang, Y., A novel aunp-based glucose oxidase mimic with enhanced activity and selectivity constructed by molecular imprinting and $o_2$-containing nanoemulsion embedding. *Adv. Mater. Interfaces* **2018,** *5* (22), 1801070.
